# A Fuzzy-Innovation-Based Adaptive Kalman Filter for Enhanced Vehicle Positioning in Dense Urban Environments

**DOI:** 10.3390/s19051142

**Published:** 2019-03-06

**Authors:** Rinara Woo, Eun-Ju Yang, Dae-Wha Seo

**Affiliations:** 1 Center for Embedded Software Technology, Kyungpook National University, 80 Daehak-ro, Buk-gu, Daegu 41566, Korea; skymin16@hotmail.com (R.W.); gong@cest.re.kr (E.-J.Y.); 2 School of Electronics Engineering, Kyungpook National University, 80 Daehak-ro, Buk-gu, Daegu 41566, Korea

**Keywords:** adaptive Kalman filter, fuzzy logic, innovation, sensor fusion

## Abstract

In this paper, a fuzzy-innovation based adaptive extended Kalman filter (FI-AKF) is proposed to improve the performance of the GNSS/INS fusion system, which is degraded due to satellite signal cutoff and attenuation and inaccurate modeling in dense urban environments. The information used for sensor fusion is obtained from real-time kinematic (RTK), micro-electro-mechanical system based inertial measumrement unit (MEMS-IMU), and on-board diagnostics (OBD). The fuzzy logic system is proposed to adaptively update the measurement covariance matrix of the RTK according to the position dilution of precision (PDOP), the number of receivable satellites, and the innovation of the extended Kalman filter (EKF). In addition, the driving state of the vehicle is defined as stop, straight run, left/right turn, and the like. To reduce the heading estimation error of the Kalman filter, the estimated heading is corrected according to the driving state. Also, the measurement covariance matrices of IMU and OBD are applied adaptively considering the characteristics of each sensor according to the driving state. In order to analyze the performance of the proposed FI-AKF positioning system in a dense urban environment, a computer simulation is performed. The proposed FI-AKF is compared to the performance of the existing extended Kalman filter and the innovation-based adaptive extended Kalman filter. In addition, we conduct a performance comparison experiment with a commercial positioning system in the field test. Through each experiment, it is confirmed that the proposed FI-AKF system has higher positioning performance than the comparison positioning systems in a dense urban environment.

## 1. Introduction

According to a standard document [1] released by the National Highway Traffic Safety Administration (NHTSA), a high accuracy of vehicle positioning is required to provide a seamless vehicle safety service. Recently, high precision positioning systems with a positioning error within a few centimeters using lidar, camera, and so on have been developed. These positioning systems are used to build high-definition maps, such as the mobile mapping system (MMS), or to develop autonomous cars. Sensors such as lidar and cameras have high resolution and accuracy, but they are used as environment recognition sensors to get the relative location. For positioning, the most standard sensor that has been used for the longest period of time is GNSS which can obtain the geometric absolute position using satellite information.

In order to improve the positioning accuracy of the GNSS with a maximum error of several tens of meters, depending on the radio receiving environment, studies have been carried out on a multi-constellation technique [2,3,4] using a dual frequency or using a correction signal such as a Differential Global Positioning System (DGPS) or RTK [5,6]. The stationary base station transmits the pseudo-range or carrier phase measurement of the received satellite as a correction message, and the receiver of the vehicle can obtain the positioning accuracy at the cm-level using the difference between the received satellite information and the correction message. In order to compensate for the distance limitations of the radio waves transmitting the correction signal to the vehicle and the limitation of the allowable distance due to the distance-dependent errors that increase as the baseline moves away, a method that generates and transmits a correction signal using a number of base stations, such as multi-reference DGPS or network RTK (N-RTK), is used [7].

However, satellite navigation systems such as GNSS and RTK have the disadvantage that they cannot determine the position properly in environments such as tunnels or elevated roads or dense urban areas, because in these environments, the satellite signals cannot be received or the satellite signals are intermittently broken or attenuated. That is why research has been conducted to reduce the positioning error of the satellite navigation system and to show the results of continuous positioning by converging with other sensors such as IMU [4,8,9,10,11,12,13].

In order to improve the performance of the sensor fusion system, it is important to select and to model the integrated filter methods considering the characteristics of each sensor. IMU is less accurate than GNSS and has drift, but it performs at a certain level without any performance change due to environmental factors. On the other hand, GNSS is accurate when the receiving environment is good, but when the receiving environment is not good, the positioning error may suddenly increase or the output may not be generated. In the GNSS/INS. positioning system, the performance of the combined positioning can be improved by increasing the weight of the GNSS when the positioning performance of the GNSS is good and decreasing the weight of the GNSS when the positioning performance of the GNSS is poor. Therefore, an adaptive Kalman filter that operates adaptively by controlling the Kalman gain directly or adjusting the process/measurement covariance matrix, which is the factor that determines the Kalman gain, has been studied [10,14,15,16].

Recently, research on the performance improvement of the GNSS/IMU fusion sensor system in dense urban environments has been carried out. Additionally, research on the design of the sensor fusion filter, such as the innovation adaptive estimation based adaptive extended Kalman filter (IAE-AKF), has been carried out [12,17,18,19].

Gao [17] proposed the novel IAE algorithm to solve the problem of estimating measurement noise uncertainty in the integrated GNSS/INS system. The singular value problem was solved and the convergence rate and estimation accuracy of the filter were improved during the existing matrix inverse operation.To this end, the filter gain matrix was indirectly modified by innovation covariance which was estimated using the regular factor.

Ding [12] proposed a new adaptive process noise scaling algorithm. The scaling factor α, which is a rough ratio between the calculated innovation covariance and predicted innovation covariance is calculated. Factor α provides the full ranges of option to tune *Q*. However, the proposed new covariance-based adaptive process noise scaling method cannot provide optimal noise allocation for each source, because it is only used to tune the magnitude of *Q*, not each element.

Zheng [20] proposed a robust adaptive unscented Kalman filter (RAUKF) to improve the accuracy of state estimation and robustness of UKF. This method includes the use of an online fault-detection mechanism to judge whether the current noise covariance needs to be updated or not. Additionally, the innovation-based method and residual-based method are used to estimate the current noise covariance of the process and measurement, respectively, if needed. The estimated values as the last noise covariance matrices and new noise covariance matrices are combined by using a weighting factor. Finally, the state estimation is modified by new noise covariance matrices and previous state estimations.

However, to be used in the positioning of vehicles running in the city, the adaptive Kalman filters based on the IAE have problems in that they do not accurately reflect a change in the driving state of the vehicle or the change of state in the place where the performance change of the satellite navigation position sensor is severe, such as urban environments. Failure to accurately model sensor characteristics in dense urban environments can reduce the performance of the filter and even cause the divergence of the filter. Therefore, in order to improve the vehicle positioning performance in a dense urban environment, it is necessary to design a sensor fusion filter that accurately models the sensor’s characteristics.

In this paper, loosely-coupled (LC) integration, which has low complexity and is an easy to use modular combination is used. Both GPS and GLONASS are used to increase the number of receivable satellites. For a better solution in a situation where the number of satellites in-view is less than four, the steering angle is used to compensate the yaw rate from the INS, and the velocity calculated from 4-wheel RPM is used to supplement the velocity from the INS. A fuzzy system is proposed to adaptively apply measurement noise covariance by position dilution of precision (PDOP), the number of receivable satellites, and the difference between the predicted position and the measurement position. Also, the heading is revised and the measurement noise covariance of IMU and OBD is applied depending on the vehicle’s driving state. We propose an adaptive EKF model that is applied depending on the fuzzy system and vehicle driving state and then evaluates the performance of it.

This paper is composed as follows. In Section 2, we describe existing research related to Kalman filtering for sensor fusion. Section 3 describes the proposed positioning system for improving the positioning performance in a dense urban environment. In Section 4, performance comparison experiments with existing fusion systems using computer simulation are described to compare the performance of the proposed sensor fusion systems. Section 5 describes the results of the actual vehicle test in the scenario constructed for the comparison of the performance of the proposed combined positioning system. Section 6 presents the conclusions about the proposed positioning system.

## 2. Related Work

### 2.1. Conventional Loosely-Coupled Kalman Filter

**Figure 1:**
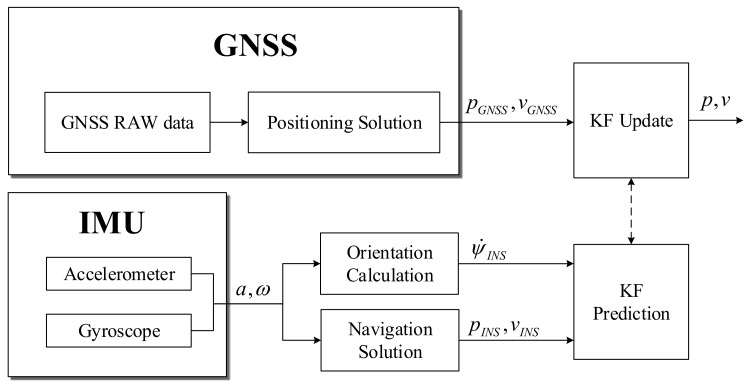
Conventional GNSS/INS loosely-coupled Kalman filter.

The conventional loose integration architecture of GNSS/INS is shown in Figure 1. The magnetometer is difficult to use in the vehicle with many ironworks due to magnetic field distortion and the difficulties of calibration at the performance levels of MEMS-IMU, although it can be used to obtain orientation. This is why only gyroscopes and accelerometers are normally used to get an orientation. The orientation, position, and velocity calculated using acceleration and the angular rate from IMU are set into the state of the Kalman filter. The Kalman filter is updated using position and velocity data calculated from GNSS.

Calculating the position and velocity using only acceleration and angular rate from IMU causes many errors, because it has first and second-order integrals. Especially in a situation where the number of receivable satellites is less than four, the positioning system cannot obtain position results with GNSS, since the positioning system has become reliant on the position result from INS, and the performance of INS can cause many errors. Consequently, the positioning system that simply fuses GNSS and MEMS-IMU may not be able to satisfy lane-level vehicle positioning. Aiding sensors or the use of the additive performance improvement method are needed to improve and compensate these drawbacks of using MEMS-IMU.

### 2.2. Innovation-Based Adaptive Estimation (IAE) for Adaptive Kalman Filtering

When modeling a Kalman filter, an error model should be developed to describe the system more accurately. It is also important to know the a priori information about the process noise and the update measurement noise in order to optimize the estimation algorithm. However, it is difficult to obtain accurate a priori information in practice. Especially in the case of GNSS/INS kinematic applications, the environment noise is not always fixed but is subject to change. In fact, wrong a priori information could decrease the performance of the Kalman filter and even cause the divergence of the filter. Especially in fusion filters using multi-sensors, the impact of challenges is extensive. Because of this, by adaptively changing noise covariance depending on the environment, the performance of the filter is improved and the robustness of it is enhanced.

Zhou et al. [10] proposed the adaptive tightly-coupled aiding (ATCA) system to remedy the disadvantage of GNSS/INS EKF. External auxiliary measurements, such as heights and headings, are added to increase the system’s observability and to provide stronger position and velocity constraints. An adaptive Kalman filter is applied to adjust the measurement noise covariance matrix in real-time based on the situation, instead of using a constant matrix, to reflect the noise characteristics accurately. Additionally, the filter is redesigned to be able to use standard tightly-coupled (TC) integration when the number of receivable satellites is more than 4, use height-aiding integration for 3, and use height/magnetic aiding integration for 2.

IAE uses the innovation sequence to estimate the system process and/or the measurement noise covariance based on the Maximum-Likelihood (ML) criterion. The innovation sequence is defined as in Equation (16), and the estimated covariance of innovation can be obtained according to [21] as follows:

(1) $${\hat{C}}_{k}=\frac{1}{N}\sum_{i=0}^{N-1}d_{k-i}d_{k-i}^{T}$$

where $d_{k}$ is the innovative sequence, *N* is the window size. The innovative sequence represents the difference between state and measurement predicted from the Kalman filter. In order to compare the theoretical value of the innovation covariance with the estimated value, the estimated value of the measurement noise covariance matrix can be obtained as follows:

(2) $${\hat{R}}_{k+1}^{-}={\hat{C}}_{k}-H\cdot P_{k}^{-}\cdot H^{T}.$$

In addition, the estimated system process noise covariance matrix can be obtained as follows, according to [21].

(3) $${\hat{Q}}_{k+1}=\frac{1}{N}\sum_{j=j_{0}}^{k}\Delta x_{j}\Delta x_{j}^{T}+P_{k}-A\cdot P_{k}\cdot A^{T}\approx K_{k}\cdot {\hat{C}}_{k}\cdot K_{k}^{T}$$

where Δx is the state correction sequence. The Kalman filter can be divergent when Q and R are unknown. To solve this problem, the proposed novel IAE-AKF can improve the stability of the filter.

However, basically, the IAE algorithm can not reflect reality because it has a moving estimation window for the innovation sequence. This is because the estimated matrix ${\hat{C}}_{k}$ is also included continuously during the window size, since the innovation vector may contain acute outliers or disturbed innovations. This can reduce the accuracy of the filter and even cause filter divergence. In order to solve the problem of IAE-AKF, more accurate modeling or additional performance enhancement technology should be considered.

## 3. Proposed Fuzzy-Innovation Based Adaptive Extended Kalman Filter (FI-AKF)

The main ideas to solve the drawbacks of conventional GNSS/INS integrated system are (1) to N-RTK not GNSS and to use additive measurements such as steering angle and velocity calculated from a 4-wheel RPM sensor to complement the estimation performance of the yaw rate and the velocity of IMU; and (2) to adopt the adaptive noise estimation to directly reflect the performance change of each sensor over a dynamic environment.

**Figure 2:**
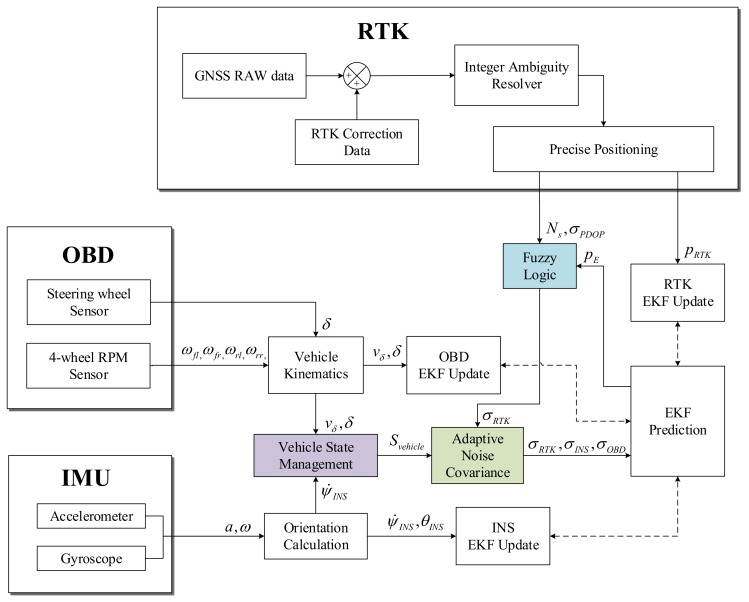
The proposed fuzzy-innovation based adaptive EKF architecture.

The proposed sensor fusion method can be further refined by (1) using the steering angle to compensate the yaw rate and using the velocity calculated from the 4-wheel sensor to substitute the velocity obtained from MEMS-IMU; (2) compensating the heading and changing the measurement noise covariance over the defined driving state; (3) using a fuzzy logic system that adaptively estimates measurement noise based on PDOP, the number of receivable satellites, and the difference between predicted and measured positions; (4) proposing an EKF model that adopts adaptively based on fuzzy logic and the driving state of the vehicle. The proposed sensor fusion system is shown in Figure 2.

### 3.1. Steering Angle Aided Yaw Rate Estimation

As mentioned in the previous section, magnetometers are hard to use in vehicles. This is why the gyroscope is used only to calculate the yaw rate generally. In this paper, information that can be obtained from on-board diagnostics II (OBDII) instead of the magnetometer is exploited to enhance the estimation accuracy of the yaw rate.

**Figure 3:**
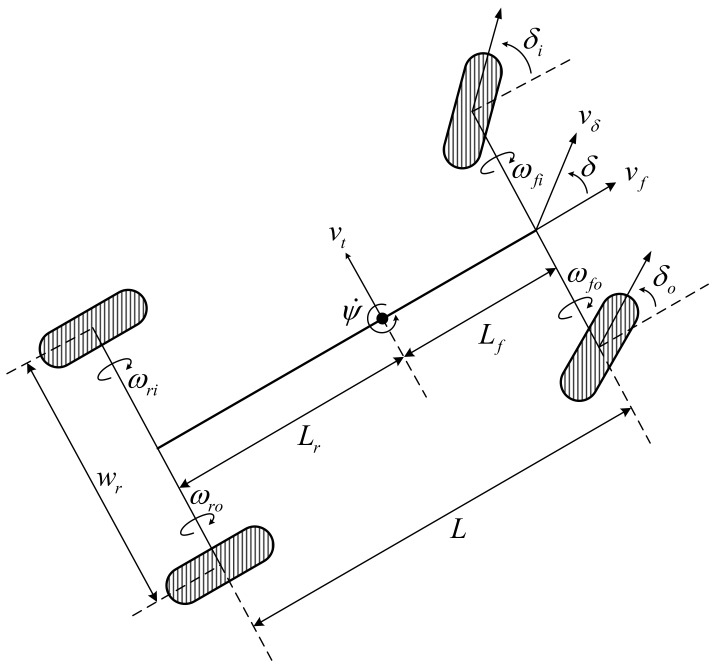
Kinematics of lateral vehicle motion.

The yaw rate is calculated from the steering angle δ and the 4-wheel RPM $\omega_{fi}$, $\omega_{fo}$, $\omega_{ri}$ and $\omega_{ro}$. In order to derive the yaw rate, the kinematics of lateral vehicle motion are shown in Figure 3. We considered a single-axle vehicle with a front-steered wheel. It was assumed that the wheels did not slip and that the center of gravity was located on the rear axle.

The velocity of each wheel $v_{\omega_{x}}$ can be written as

(4) $$v_{\omega_{x}}=\omega_{x}\cdot f_{d}\cdot w_{c},$$

where $\omega_{x}$ is 4-wheel RPM, $f_{d}$ is the frequency divider, and $w_{c}$ is the wheel circumference.

The wheel path velocity $v_{\delta }$ and forward velocity $v_{f}$ are given by

(5) $$v_{\delta }=\left(v_{\omega_{fi}}+v_{\omega_{fo}}\right)/2,$$

(6) $$v_{f}=\left(v_{\omega_{ri}}+v_{\omega_{ro}}\right)/2.$$

The $v_{f}$ can also be expressed as

(7) $$v_{f}=v_{\delta }\cdot \cos\delta .$$

The yaw rate obtained from $v_{\delta }$, the steering angle, and the wheel base *L* can be expressed as

(8) $$\dot{\psi }=v_{\delta }\cdot \sin\delta /L.$$

The data rate of the steering angular sensor and 4-wheel sensor is irregular (30–50 Hz), and the data rate of IMU is 100 Hz. The all average yaw rates calculated from IMU and steering angular sensor are quite similar. The yaw rate of the IMU fluctuates but the data rate is higher than the steering angle. The yaw rate calculated from the steering angle sensor has no variation. However, since the data rate of the steering angle is relatively lower than that of the IMU, the accuracy of the caclulated yaw rate is low. In IMU, the gyro drift exists due to the sensor characteristics. To reduce this error, initialization and calibration using aiding sensors are needed. Particularly, in the case of MEMS-IMU which has low accuracy, a solution for this is more needed. Sometimes the steering angle sensor has a non-zero value due to an architectural problem when the handle is in the center position. Consequently, both sensors must be used together to obtain enhanced accuracy by compensating for each other. In this paper, the steering angle is used as the state variable, and the yaw rate of IMU is used as the measurement variable to fuse these in EKF.

(9) $$p_{INS/OBD}^{n}=p_{INS/OBD}^{n}+D\cdot v_{\delta ,k-1}^{b}\cdot \Delta t,$$

where *D* is the matrix to transform velocity components to the derivative of geographic coordinates and has the expression:

(10) $$D=\left[\begin{array}{c} \cos\delta \cdot \cos\psi \cdot \cos\theta \\ \cos\delta \cdot \sin\psi \cdot \cos\theta \\ \sin\theta \end{array}\right]$$

where θ is the inclination of the vehicle.

### 3.2. Driving State Classification

To predict the position of vehicle at the next epoch, the heading information of the vehicle as well as the speed information of the vehicle is very important. By fusing the heading calculated with the variance in position using high precision RTK and the heading calculated from IMU, a more accurate heading may be acquired than that acquired using GNSS/INS. However, the fused heading always cannot be expected to be accurate because of the limited number of satellites in view or the error of IMU. Despite the vehicle being stopped, the output of the satellite navigation system changes continuously, and the calculated heading changes also. Consequently, when the vehicle is stopped, the heading calculated from IMU is more accurate than RTK. The position is modified with the RTK condition using the properties whereby the longer the measure is, the more converge to the true value there is while the vehicle is stationary. As described in the previous section, the offset of the steering angle is calibrated using the yaw rate calculated from IMU when the vehicle is going straight. Consequently, we need to define whether the vehicle is stationary, going straight, or turning and whether the satellite navigation system is reliable or not. In this paper, the driving state of the vehicle is defined as stop, start, straight run, left/right turn, and waiting. The vehicle driving states are categorized based on the velocity, yaw rate, and state holding times. The reliability of each sensor is reflected differently depending on the vehicle driving state.

When the vehicle is stationary, the satellite navigation positioning system, which has a basic probability error, outputs a varying value, although there is some difference depending on the radio reception environment. Therefore, we assume that the positioning reliability of the satellite navigation system is relatively low at the stopping time, and we apply it. As mentioned above, the heading value obtained based on the position change significantly improves the accuracy of the straight running, so that the heading value can be readjusted when the straight running is clearly determined. The position and yaw rate at the next time point can be predicted based on the dynamics of the vehicle at the time that the vehicle is going straight forward or turning left/right. In the case of the satellite navigation system and inertial navigation system, innovation can be used as a criterion to judge the reliability of the sensor value, because it can have a high level of error according to the situation.

When the velocity of the vehicle is slower than 0.2 m/s, the vehicle is defined as being in a stationary state. When in a stationary state, the positioning results of RTK are converged by dozens or hundreds of centimeters, as long as the signal reception of the satellites is not bad. In this state, consequently, it is estimated that the positioning result of RTK is extremely reliable. But, the heading does not change no matter how reliable the positioning result of RTK is. When the current state is stationary state and the velocity of the vehicle is faster than 0.2 m/s and the yaw rate is less threshold, the vehicle is defined as in a start-up straight state. In this state, steering angle is calibrated by the yaw rate calculated from IMU. If the start-up straight state hold for 1 second, the vehicle is defined as in a straight state. When the current state is stationary and the velocity of the vehicle is faster than 0.2 m/s and the yaw rate is over the threshold, the vehicle is defined as being in a start-up turning left or a start-up turning right state. If the start-up turning left/right state holds for 1 s, the vehicle is defined as being in a turning left/right state. When the current is in a straight or turning left/right state, the reliability of each sensor is determined by the dynamics of vehicles in each driving state. Consequently, the criteria for determining the driving state and the algorithm policy in each driving state are shown in Table 1.

### 3.3. Fuzzy Logic for RTK Reliability

**Figure 4:**
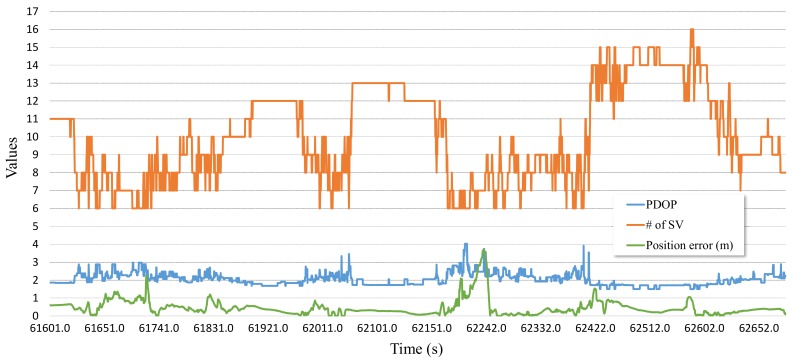
Relation graph between PDOP, number of satellites, and innovation.

While the satellite navigation system can acquire the absolute position, unlike most the other sensors, there is a drawback in that the reliability of it is poor in situations where the number of satellites in view is not enough, such as in dense urban areas and below overpasses or tunnels. With RTK, which is a high-precision positioning device, while the positioning error is of a few centimeters in good satellite receivable areas, it is more than a few meters in bad ones. Consequently, good reliability of the satellite navigation positioning results over the satellite receivable area is needed to reflect sensor fusion. The PDOP of satellites and the number of satellites in view are used for the purpose of determining how reliable the RTK positioning results is. However, PDOP and the error over the number of satellites in view are relevant, but these have nonlinearity, as shown in Figure 4. In this paper, consequently, the fuzzy logic system that is suited to deal with objects including inaccuracy and nonlinearity is used to evaluate the reliability of the positioning error in the satellite navigation.

The positioning result of RTK is very reliable when positioning environments are not in extremely bad satellite receivable areas. The difference between the expected value of fusion results and the measured value of satellite navigation positioning is added as a criterion for determining the reliability of RTK. The Mamdani method [22] is used for fuzzy logic.

If the surrounding environment is not only good for satellite positioning but also extremely bad, RTK satellite navigation positioning reliability is high, so the expected value of convergence result is added as a criterion to determine the reliability of RTK satellite navigation.

**Figure 5:**
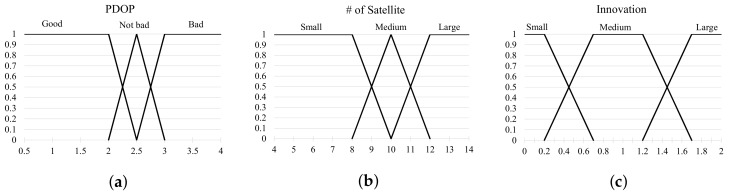
Fuzzy membership function. (**a**) PDOP; (**b**) The number of satellite; (**c**) Innovation.

Figure 5 shows the fuzzy membership function for the PDOP, the number of visible satellites, and the innovation of the EKF. The first controller, which uses PDOP and the number of visible satellites, is used as the system input, and the second controller inputs a distance error between an output of the first controller and an expected value. The fuzzy control rules for these controllers are shown in Table 2. The inputs of the fuzzy control rule 1 are PDOP and the number of visible satellites, and the output *A* of it is expressed as either small, medium, or large. Fuzzy logic controller 1 uses the following nine rules:

**If** the number of receivable satellites is small **and** the PDOP is bad, **then** A is bad.

**If** the number of receivable satellites is large **and** the PDOP is not bad, **then** A is good.

The input of the fuzzy control rule 2 is the output *A* of fuzzy control rule 1 and the innovation, and the output is expressed as the reliability of the satellite navigation positioning result σ. If the output of the fuzzy logic controller is bad, the noise covariance of the satellite navigation positioning results is set to a large value. The sample rule is as follows:

**If** A is bad **and** the innovation is large, **then** σ is medium.

The output of the fuzzy control rule 2, σ, is represented as very small, small, medium, or large and is subjected to a fuzzy process to obtain numerical data from each output. The center of average method is used for the defuzzifier.

### 3.4. Proposed Extended Kalman Filter Model

The state vector of the proposed EKF is as follows:

(11) $$x_{k}={\left[\begin{array}{ccccccccc} \psi_{k} & {\dot{\psi }}_{k} & \delta_{k} & v_{f,k} & v_{\delta ,k} & \theta_{k} & p_{E,k} & p_{N,k} & p_{U,k} \end{array}\right]}^{T},$$

where $\psi_{k}$ is the heading in the body frame, ${\dot{\psi }}_{k}$ is the yaw rate in the body frame, $\delta_{k}$ is the steering angle in the body frame, $v_{f,k}$ is the forward velocity in the body frame, $v_{\delta ,k}$ is the wheel path velocity in the body frame, $\theta_{k}$ is the inclination in the body frame, ${\left[\begin{array}{ccc} p_{E,k} & p_{N,k} & p_{U,k} \end{array}\right]}^{T}$ is the local navigation frame vector in the ENU frame, and subscript *k* is the time index.

The system state vector ${\hat{x}}^{-}$ and the covariance matrix $P^{-}$ are expressed as follows:

(12) $${\hat{x}}_{k}^{-}=f_{k-1}\left({\hat{x}}_{k-1},w_{k-1}\right),w_{k}∼\left(0,Q_{k}\right),$$

(13) $$P_{k}^{-}=F_{k-1}\cdot P_{k-1}\cdot F_{k-1}^{T}+Q_{k-1},$$

where $f\left(\cdot \right)$ is the state transition matrix, and the partial derivative matrix *F* is as follows:

(14) $$f_{k-1}\left(x_{k-1},w_{k-1}\right)=\left[\begin{array}{c} \psi_{k-1}\\ {\dot{\psi }}_{k-1}\\ \delta_{k-1}\\ v_{k-1}\\ v_{\delta ,k-1}\\ \theta_{k-1}\\ p_{E,k-1}\\ p_{N,k-1}\\ p_{U,k-1} \end{array}\right]=\left[\begin{array}{c} \psi_{k-1}+{\dot{\psi }}_{k-1}\cdot \Delta t\\ v_{k-1}/L\\ \delta_{k-1}\\ v_{\delta ,k-1}\cdot \sin\delta \\ v_{\delta ,k-1}\\ \theta_{k-1}\\ p_{E,k-1}+v_{\delta ,k-1}\cdot \Delta t\cdot \cos\delta \cdot \cos\psi \cdot \cos\theta \\ p_{N,k-1}+v_{\delta ,k-1}\cdot \Delta t\cdot \cos\delta \cdot \sin\psi \cdot \cos\theta \\ p_{U,k-1}+v_{\delta ,k-1}\cdot \Delta t\cdot \sin\theta \end{array}\right]+w_{k-1},$$

(15) $$F_{k-1}={\left[\begin{array}{ccccccccc} 1 & \Delta t & 0 & 0 & 0 & 0 & -v_{\delta ,k-1}\cdot \Delta t\cdot \cos\delta \cdot \sin\psi \cdot \cos\theta & v_{\delta ,k-1}\cdot \Delta t\cdot \cos\delta \cdot \cos\psi \cdot \cos\theta & 0\\ 0 & 1 & 0 & 0 & 0 & 0 & 0 & 0 & 0\\ 0 & 0 & 1 & v_{\delta ,k-1}\cdot \cos\delta & 0 & 0 & -v_{\delta ,k-1}\cdot \Delta t\cdot \sin\delta \cdot \cos\psi \cdot \cos\theta & -v_{\delta ,k-1}\cdot \Delta t\cdot \sin\delta \cdot \sin\psi \cdot \cos\theta & 0\\ 0 & 1/L & 0 & 1 & 0 & 0 & 0 & 0 & 0\\ 0 & 0 & 0 & \sin\delta & 1 & 0 & \Delta t\cdot \cos\delta \cdot \cos\psi \cdot \cos\theta & \Delta t\cdot \cos\delta \cdot \sin\psi \cdot \cos\theta & \Delta t\cdot \sin\theta \\ 0 & 0 & 0 & 0 & 0 & 1 & -v_{\delta ,k-1}\cdot \Delta t\cdot \cos\delta \cdot \cos\psi \cdot \sin\theta & -v_{\delta ,k-1}\cdot \Delta t\cdot \cos\delta \cdot \sin\psi \cdot \sin\theta & v_{\delta ,k-1}\cdot \Delta t\cdot \cos\theta \\ 0 & 0 & 0 & 0 & 0 & 0 & 1 & 0 & 0\\ 0 & 0 & 0 & 0 & 0 & 0 & 0 & 1 & 0\\ 0 & 0 & 0 & 0 & 0 & 0 & 0 & 0 & 1 \end{array}\right]}^{T}.$$

The state vector $\hat{x_{k}}$, its covariance $P_{k}$, and the Kalman gain $K_{k}$ estimated in the measurement process are as follows:

(16) $${\hat{d}}_{k}^{-}=z_{k}-h\left({\hat{x}}_{k}^{-}\right),$$

(17) $$S_{k}=H\cdot P_{k}^{-}\cdot H^{T}+R_{k},$$

(18) $$K_{k}=P_{k}^{-}\cdot H^{T}\cdot S_{k}^{-1},$$

(19) $${\hat{x}}_{k}={\hat{x}}_{k}^{-}+K_{k}\cdot {\tilde{d}}_{k}^{-},$$

(20) $$P_{k}=\left(I-K_{k}\cdot H\right)\cdot P_{k}^{-},$$

where $d_{k}^{-}$ is the innovation sequence, $S_{k}$ is the innovation covariance matrix $P^{-}$ is the covariance matirx of the predicted state, *H* is the measurement matrix, and *R* is the measurement noise covariance matrix.

The state vector $x_{k}$ and the covariance matirx of the measurement state $P_{k}$ are updated with the Kalman gain, the predicted state ${\hat{x}}_{k}$, the measurement vector $z_{k}$, and ${P_{k}}^{-}$. $z_{k}$ of each sensor is modeled on the linear function of ${\hat{x_{k}}}^{-}$.

For the measurement update by RTK data, the measurement vector $z_{RTK}$, the measurement model matrix $H_{RTK}$, and the measurement noise covariance matrix $R_{RTK}$ are as follows:

(21) $$z_{RTK,k}={\left[\begin{array}{ccc} p_{E,k}^{RTK} & p_{N,k}^{RTK} & p_{U,k}^{RTK} \end{array}\right]}^{T},$$

(22) $$H_{RTK}=diag\left(\begin{array}{ccccccccc} 0 & 0 & 0 & 0 & 0 & 0 & 1 & 1 & 1 \end{array}\right),$$

(23) $$R_{RTK}=diag\left(\begin{array}{ccc} \sigma_{{RTK}_{N}}^{2} & \sigma_{RTK_{E}}^{2} & \sigma_{RTK_{D}}^{2} \end{array}\right),$$

where $z_{RTK}$ includes the position vector ${\left[\begin{array}{ccc} p_{E,k}^{RTK}, & p_{N,k}^{RTK}, & p_{U,k}^{RTK} \end{array}\right]}^{T}$. The position output of RTK is converted from geodetic coordinates to ENU coordinates. $\sigma_{RTK_{N}}$,$\sigma_{RTK_{E}}$ and $\sigma_{RTK_{D}}$ are the deviations of the RTK position vector that are to be specified by the output of fuzzy logic.

When fusing the position of the IMU/OBD with the position of the RTK measurement, a lever-arm offset occurs due to the positioning error between the center of the installed IMU sensor and the center of the RTK antenna. This leads to a positioning error and needs to be corrected. The lever-arm correction method in n-frame can be expressed as follows:

(24) $$r_{RTK}^{n}=r_{IMU/OBD}^{n}+R_{b}^{n}l_{RTK}^{b}$$

where, $r_{RTK}^{n}$ and $r_{IMU/OBD}^{n}$ is position coordinates in the n-frame for the RTK rover receiver and the IMU/OBD center, $R_{b}^{n}$ is the rotation matrix fromm the b-frame to the n-frame, and $l_{RTK}^{b}$ is the lever-arm offset vector in the b-frame.

For the measurement update by INS data, the measurement vector $z_{INS}$, measurement model matrix $H_{INS}$, and measurement noise covariance matrix $R_{INS}$ are as follows:

(25) $$z_{INS,k}={\left[\begin{array}{cc} {\dot{\psi }}_{k}^{INS} & \theta_{k}^{INS} \end{array}\right]}^{T},$$

(26) $$H_{INS}=diag\left(\begin{array}{ccccccccc} 0 & 1 & 0 & 0 & 0 & 1 & 0 & 0 & 0 \end{array}\right),$$

(27) $$R_{INS}=diag\left(\begin{array}{cc} \sigma_{INS}^{2} & \sigma_{INS}^{2} \end{array}\right),$$

where $z_{INS}$ includes the yaw rate ${\dot{\psi }}^{INS}$ and the inclination $\theta^{INS}$ calculated from the accelerator and the gyroscope. $\sigma_{INS}$ is the deviation of the INS orientation output that is specified by the output of the adaptive noise estimation. For the measurement update by OBDII data, the measurement vector $z_{OBD}$, measurement model matrix $H_{OBD}$ and measurement noise covariance matrix $R_{OBD}$ are as follows:

(28) $$z_{OBD,k}={\left[\begin{array}{ccc} \delta_{k} & v_{f,k} & v_{\delta ,k} \end{array}\right]}^{T},$$

(29) $$H_{OBD}=diag\left(\begin{array}{ccccccccc} 0 & 0 & 1 & 1 & 1 & 0 & 0 & 0 & 0 \end{array}\right),$$

(30) $$R_{OBD}=diag\left(\begin{array}{ccc} \sigma_{OBD}^{2} & \sigma_{v_{f}}^{2} & \sigma_{v_{\delta }}^{2} \end{array}\right)$$

where $z_{OBD}$ includes the steering angle δ, the forward velocity $v_{f}$, and the wheel path velocity $v_{\delta }$. $\sigma_{OBD}$ is the deviation of the steering angle δ that is specified by the output of the adaptive noise estimation. $\sigma_{v_{f}}^{2}$ and $\sigma_{v_{\delta }}^{2}$ are the variances of velocities $v_{f}$ and $v_{\delta }$, respectively.

## 4. Simulations

This section describes the simulation environment and performance comparison results used for the sensor fusion systems EKF, IAE-AKF and the proposed FI-AKF described in Chapters 2 and 3.

**Figure 6:**
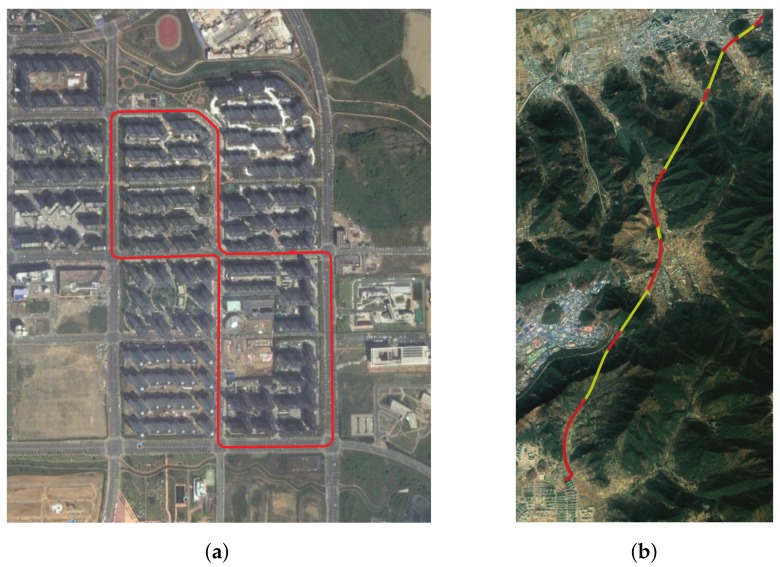
Test trajectory. (**a**) Scenario I—dense urban area; (**b**) Scenario II—tunnels (red—roads, yellow—tunnels).

The input of the simulation model is the RTK observables acquired through the N-RTK receiver, the IMU raw data acquired through the MPU-9250, and the steering angle and 4-wheel sensor data obtained through a vehicle. In this experiment, the Flach Korrektur Parameter (FKP) was used in the N-RTK method. The long-term evolution (LTE) router was used to connect to the N-RTK server for a kinematic survey of the vehicle. After mounting the data collect device equipped with the above sensors on the vehicle, the data was obtained by circling the section shown in Figure 6a. RTK positioning, INS orientation and vehicle kinematics were implemented in the MATLAB environment. RTK positioning was implemented based on RTKLIB and was designed to output the positioning results in each epoch rather than filtering according to dynamics. Therefore, the output result in a situation where the ambiguity constant determination rate was lowered could have had a somewhat higher positioning error. The MEMS-IMU sensor used was of consumer grade and its specifications are the same as listed in Table 3.

In order to derive the positioning error of the experimental group, the position was corrected with the precision map using the RTK/IMU/OBD fusion result as a reference. Since the experiment site is an environment in which a high-definition map is constructed, a reference was made using it. With high-definition map information, you can find links that represent each driving lane’s centerline. The position information obtained while traveling along the links was then matched with the link through subsequent correction. Errors in travel and high-definition map can occur. Therefore, the expected positioning error of the reference was within 20 cm.

For the performance analysis of each positioning system, the average positioning error, the standard deviation, the 95th percentile and the maximum value of the positioning error according to the time interval were used as the metrics by referring to the definition defined in the European telecommunications standards institute (ETSI) standard [23].

### 4.1. Conventional EKF

**Figure 7:**
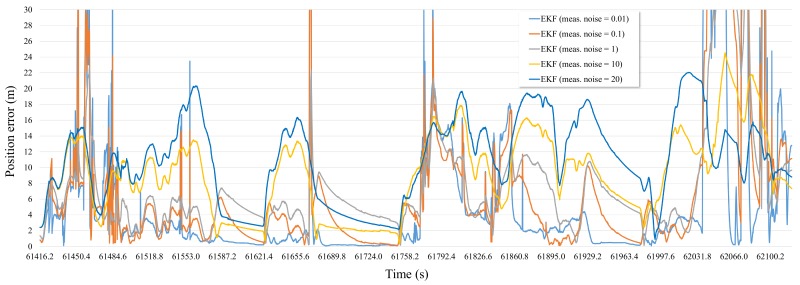
The positioning error of EKF with measurement noise variation in the simulation with raw sensor data.

To simulate the performance of the RTK/IMU/OBD positioning system using the EKF, a simulation was performed. The Figure 7 shows the positioning error of EKF when the measurement noise of RTK was fixed to 0.01, 0.1, 1, 5, 10, and 20 m, respectively.

In RTK/IMU/OBD positioning system using the EKF, each sensor data are fused as the value of RTK measurement noise becomes smaller, the reliability of the value of RTK becomes higher. And each sensor data are fused as the value of RTK measurement noise becomes larger, the reliability of the value of IMU/OBD becomes higher. The positioning error of the EKF with the RTK measurement noise of 0.01 m shows that the deviation of the positioning error was large due to the dense urban environment. In a good reception environment, it can be seen that the positioning error was less than about 1 m. But, when the reception condition of the satellite was poor due to obstacles such as buildings and trees, the positioning error was close to 30 m. The graph with measurement noise of 20 m shows that the deviation of the positioning error was smaller than the graph with measurement noise of 0.01 m, but generally the positioning error was high. This is because the measurement values of INS and OBD are more reliable than those of RTK when the measurement noise is 20 m.

**Figure 8:**
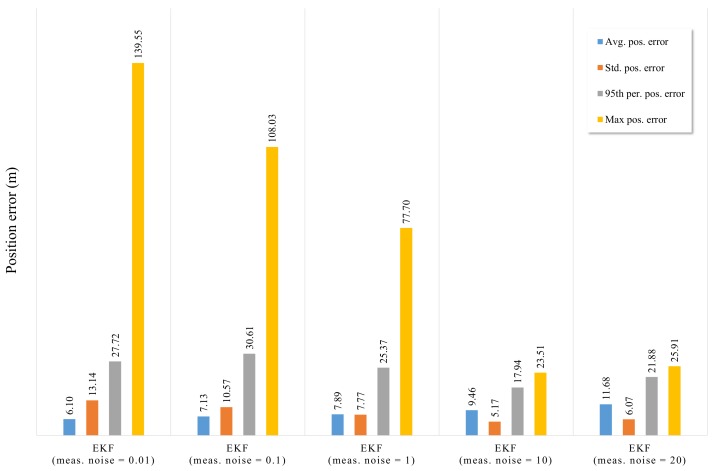
Bar graph showing the average, standard deviation, 95th percentile, and maximum error for the position of EKF with variable measurement noise covariance in the simulation with raw sensor data.

The average, standard, 95th percentile, and maximum positioning errors for the Figure 7 are shown in the Figure 8. As a result, it can be seen that the average position error of EKF with the RTK measurement noise of 0.01 m has better performance than others. However, standard deviation, 95th percentile and maximum position error of EKF with the RTK measurement noise of 0.01 m have relatively high value. As Figure 7 has shown, This is because RTK measurement has a high positioning error probability when the reception condition of the satellite was poor due to obstacles such as buildings and trees. Furthermore, it can be expected that by adjusting the value of the measurement noise matrix according to the interval where the performance of the RTK varies greatly, it can be improved.

### 4.2. IAE-Based Adaptive Extended Kalman Filter (IAE-AKF)

**Figure 9:**
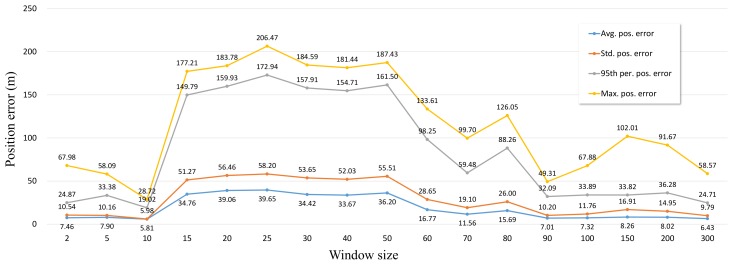
Line graph showing the average, standard deviation, 95th percentile, and maximum error for the position of IAE-AKF with a variable window size in the simulation with raw sensor data.

IAE-AKF is the adaptive Kalman filter that changes the measurement covariance matrix according to Equation (1). As shown in Equation (1), averaging is performed with a window size, which causes a performance change. We measured the positioning error of IAE-AKF by changing the window size to investigate the performance change of IAE-AKF according to the window size. The IAE-AKF positioning error results according to the IAE window size are shown in Figure 9. $\sigma_{Q}$ was set to 0.001 m, $\sigma_{INS}$ was set to 0.1 m, and $\sigma_{OBD}$ was set to 0.01 m. The IAE window size was set to 2, 5, 10, …, 200.

The fluctuation in the positioning error is large as the size changed when the window size is small, and the fluctuation of the positioning error based on the size is small when the window size is large, as seen from Figure 9. As the window size grows, more innovation is averaged, so even if the innovation is changed greatly, the change of performance is not big because the change in *R* is not large. The location where the satellite signal of RTK is not good and the location where the INS error is high are different from each other. In the situation where the RTK positioning result fluctuates due to the RTK satellite reception condition not being good, the innovation value is high, but because of the averaging, the result of RTK positioning at that time is reflected as a high weight, and the overall positioning error becomes large. On the other hand, when the stable RTK satellite reception state is established, the RTK positioning result is reflected as a low weight, because the previous RTK satellite reception state is not good. However, if the INS error is large, the overall positioning error may become large. In the experimental section, the average positioning error is significantly changed according to the value of averaging, because there are many sections with poor satellite reception and rotation sections. Even if an optimal averaging value is obtained based on this criterion, it can not be said that an optimal value will be derived in another scenario. Therefore, sensor fusion with innovation alone can obtain better results than conventional EKF, but it seems difficult to derive optimal fusion results.

### 4.3. Proposed Fuzzy-Innovation-Based Adaptive Extended Kalman Filter (FI-AKF)

**Figure 10:**
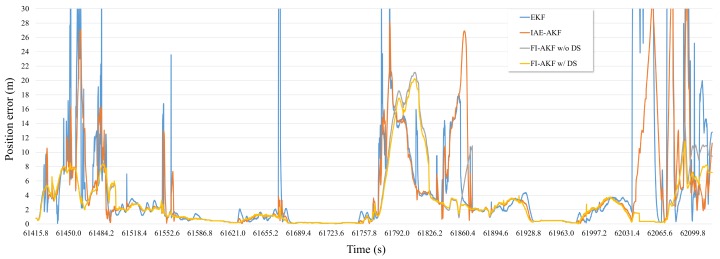
The positioning error of EKF, IAE-AKF, FI-AKF w/o DS, and FI-AKF w/DS in the simulation with raw sensor data.

A simulation was performed to assess the FI-AKF proposed to improve the performance of the sensor fusion filter using RTK, MEMS-IMU, and OBD information in dense urban environments. To analyze the performance of the proposed FI-AKF, we performed a performance evaluation by distinguishing between driving-state (w/DS) and non-driving-state (w/o DS) applications. The results of the simulation performance analysis are shown in Figure 10, together with the results of the positioning error of each filter to compare with the results of the previous EKF and IAE-AKF experiments.

As can be seen from Figure 10, FI-AKF w/o DS and FI-AKF w/DS had relatively low positioning errors compared to EKF and IAE-AKF in the initial section where the RTK positioning error increases. Especially in the region where the positioning error of the RTK rapidly decreased after 61,527.8 s, the FI-AKFs converged to the low positioning error faster than other comparative models. This means that FI-AKFs responds more quickly to changes in the performance of RTK than other comparative models, and it is also set to a more appropriate measurement covariance depending on the situation. The following region also showed a relatively low positioning error compared to EKF and IAE-AKF. The positioning errors of FI-AKF w/o DS and FI-AKF w/DS had generally similar values, and a large difference was observed in the latter half of the experiment.

**Figure 11:**
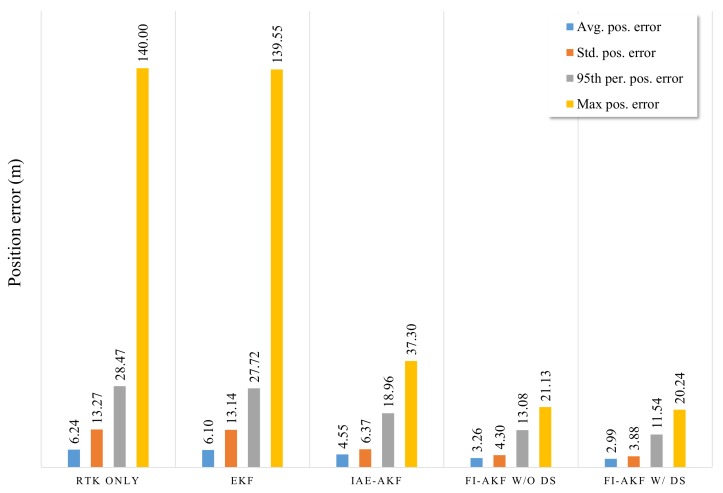
Bar graph showing the average, standard deviation, 95th percentile, and maximum error for the position of EKF, IAE-AKF, FI-AKF w/o DS, and FI-AKF w/DS in the simulation with raw sensor data.

The average, standard, 95th percentile, and maximum positioning errors for the Figure 10 are shown in Figure 11.

The results of the positioning errors of EKF and IAE-AKF are the result of measurement noise of 0.01 m and process noise of 0.1 m, which were determined in previous experimental results. The results of the FI-AKF w/o DS show that both the mean and maximum positioning errors are reduced compared to the EKF and IAE-AKF results. Using the driving state method, we can see that the average error was about 10% less than when it was not in use.

## 5. Real-Time Experimental Evaluations of FI-AKF with Commercial Positioning Systems

### 5.1. Experiment Setup

**Figure 12:**
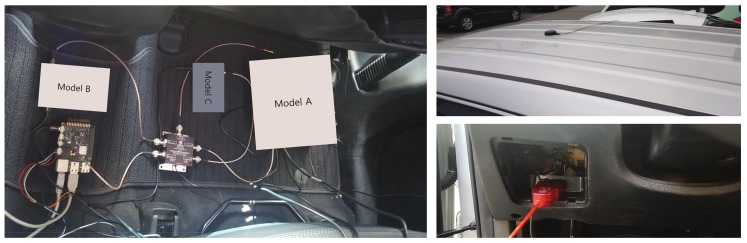
Test environments for precise positioning.

In order to evaluate the performance of the proposed FI-AKF-based real-time positioning system, we compared it with the conventional commercial positioning system in a dense urban environment and in a highway environment including tunnels. Satellite signals input to each positioning system were received by one antenna and distributed through a splitter. The proposed FI-AKF based positioning system was implemented on Raspberry PI. In order to evaluate the performance of each positioning system, references were obtained as detailed in chapter 4. We constructed a field test environment by installing a positioning system in the car, as shown in Figure 12. The scenarios used in the real-time positioning performance comparison experiment are shown in Figure 6a,b.

### 5.2. Real-Time Experimental Results in Dense Urban Environments

**Figure 13:**
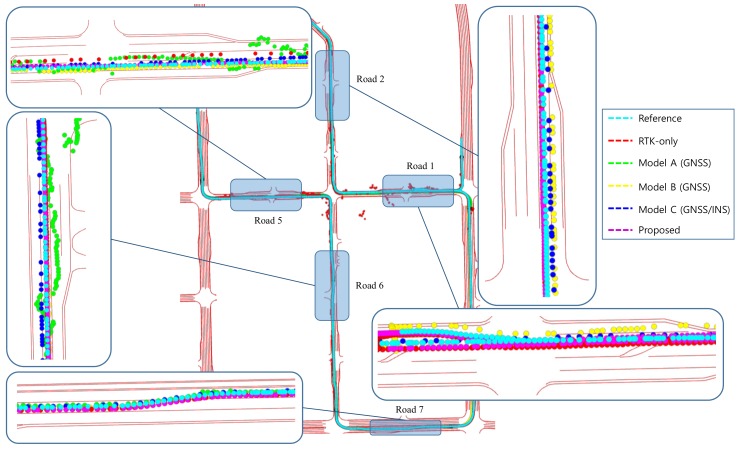
Positioning results for scenario 1.

In scenario 1, the positioning results of roads 1, 2, 5, and 6 surrounded by high-rise apartments on each side and road 7 with high-rise apartments on one side are enlarged and shown in Figure 13. When most of the surrounding area was blocked by high-rise apartments, it can be seen that the positioning values were dispersed more than the reference values. In cases where the road was surrounded by a high-rise apartment, when the number of receivable satellites was small, a large positioning error occurred or positioning results were not obtained.

The positioning result of model A is represented by red dots. Model A used less satellites than the other models to position in a situation where the number of satellites in view was not enough. Additionally, model A only output the positioning result at that epoch, because it does not have a dead-reckoning (DR) function. Consequently, model A had a higher positioning error in situations where the number of satellites in view was not enough. Although model B also does not have IMU inside the module, it output a positioning result that reflected the dynamics due to the DR function in situations where the number of satellites in view was not enough. When the previous positioning result was good and the driving state was straight, without INS, which provides accurate directional information, DR showed a relatively low positioning error. On the other hand, when the previous positioning result was bad, DR had a high positioning error, because it is subject to cumulative errors. Model C showed smooth positioning results due to being aided by INS, unlike model A. In dense urban environments, such as scenario 1, because the error of satellite navigation system increases, sometimes the positioning result appears on the other lane. Because the RTK and the proposed system use the correction signals to reduce the positioning error, the positioning error of those is lower than that of other modules using GNSS. However, for road 6, while the RTK result was also far different from the reference, there was no significant difference between the result of the proposed system and the reference.

**Figure 14:**
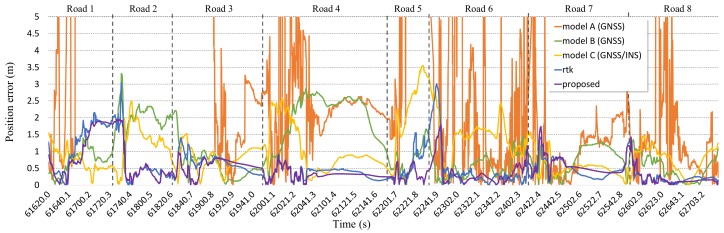
Line graph showing the positioning errors of models A, B, C, and the FI-AKF system in a dense urban scenario.

The acquired positioning error on the time axis is shown in Figure 14. The positioning error of model A was markedly different between good satellite receivable areas and bad ones. In bad satellite receivable areas, its error is as high as 5 m, and the error deviation is high. The positioning error of the others was represented by a smooth curve form because they contain DR.

**Figure 15:**
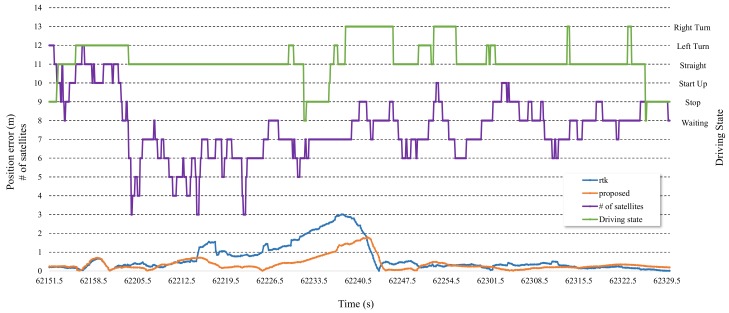
Positioning error between RTK and the proposed scheme as number of satellites and driving state.

The average and maximum position error and lane-level accuracy (LLA) for each section are shown in Table 4. As shown in Figure 14 and Table 4, the maximum positioning error was more than a few meters sometimes. At this point, the positioning result occurred in a different lane from the driving lane. In particular, it was hard to satisfy LLA above 90% in general, except in roads 7 and 8 where there was a relatively good environment to receive the satellite signals. Model A to C have the stretch of roads where the maximum positioning error was above 3.3 m and the LLA was low. The positioning result of RTK showed an average positioning error of below 0.54 and an LLA of above 90%. However, the maximum positioning error was above 2 to 3 meters and the LLA was below 90% in roads 1 to 2 and 5 to 6 where the number of receivable satellites was below 6. The positioning result of the proposed system also showed that maximum positioning error was high in roads 1 to 2 and 5 to 6 which have worse environments to receive the satellite signals. However, the maximum positioning error was over 1 m lower than that of RTK. Additionally, the maximum positioning error was under 2 meters for all roads. Also, LLA was above 95% for all roads except roads 1 and 2. The positioning errors of RTK and the proposed system are shown in Figure 15 during some periods to precisely analyze the positioning performance of the proposed system.

Figure 15 shows the number of receivable satellites, the driving state, and the positioning error of RTK and the proposed system. During this period, the vehicle started, turned left, went straight, then stopped for a while, and then drove and stopped. The number of receivable satellites was high, 10 to 12, in the early period, and it was very low, 3 to 6, in the middle period. It was 6 to 10 in the late period. The positioning error of the proposed system was nearly identical to that of RTK because the proposed adaptive algorithm adds weight to satellite positioning. For the rest of period, the positioning error increased due to the smaller number of receivable satellites. The positioning error of the proposed system was up to over 1.5 meters lower than that of RTK while the number of receivable satellites was low.

### 5.3. Real-Time Experimental Results in the Highway with Tunnel Environment

**Figure 16:**
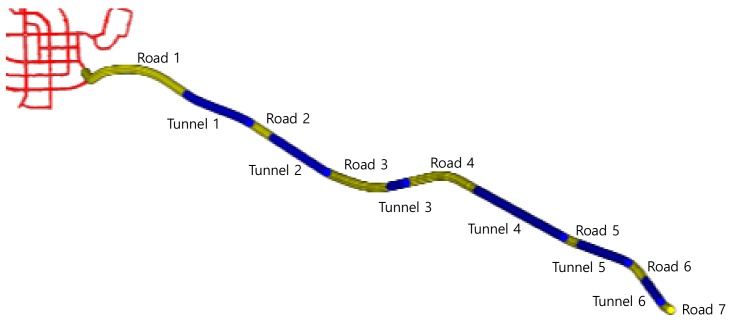
Trajectory of the true position (yellow line—roads, blue line—tunnels).

Figure 16 shows the trajectory for the roads including six tunnels. Because the positioning modules without DR function do not produce an output while going through tunnels, the measurement modules were compared in roads and tunnels. Because the positioning result of RTK cannot be gained in tunnels, and it is similar to that of the proposed system in roads, in scenario 2, all measurement modules except RTK were compared.

**Figure 17:**
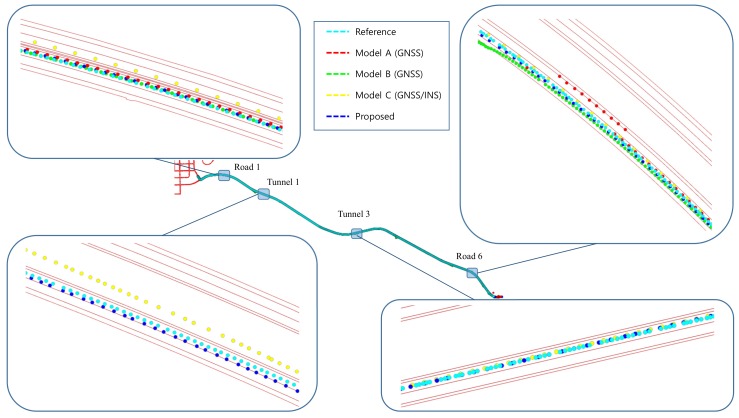
Positioning results for scenario 2.

Figure 17 shows the positioning results of proposed algorithm and compares modules in some sections. On the whole, the position results did not departed from the reference. The position of model C outside of the lane was caused by the error that appeared when cornering in beginning periods. The positioning error in tunnel 1 was also large because the signal entered the tunnel without any correction. The satellite signals are disconnected and reconnected as they enter and exit the tunnel. In this process, the error caused by fewer connected satellites was sustained until the positioning system stabilized by receiving more satellite signals. The red dots outside of the lane indicate the results of this process and show a positioning error of over 5 meters from the reference.

**Figure 18:**
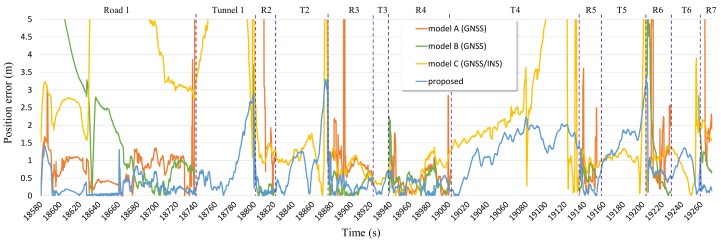
Line graph showing the positioning error of models A, B, C, and the FI-AKF system in the tunnel scenario.

The positioning error of each module over time is shown in Figure 18. The deviation of the positioning error in the scenario involving roads in open sky conditions, unlike scenario 1, was smaller than that in scenario 1. The results of all modules on the roads are shown. Additionally, the results of model C and proposed system in tunnels are shown. In road 1, there is a section that goes outside the urban area that includes the national highway. That is why the positioning error is relatively large as first and get smaller. In all roads after road 1, there was generally a lower positioning error than that in tunnels. In tunnels, because the satellite signals are not received and there is sensor drift, the positioning error gradually increased over time. The satellite signals are not received as the vehicle passes through the tunnel, and then, if the vehicle goes onto the road, it can receive the satellite signals. At this point, time is needed for stable results to occur using a large number of satellite signals. Consequently, on the road soon after the tunnel, the positioning error is relatively large for a period of time.

The average and maximum position error and lane-level accuracy for each section are shown in Table 5 and Table 6. The positioning results on the roads without tunnels are represented in Table 5. The results of models B and C are similar to those in scenario 1. The results of the proposed system demonstrate that the average positioning error has a low value of about 0.34 m in good satellite receivable areas. Besides, LLA has a higher value of 99.42%.

Table 6 shows the positioning results in tunnels. Model C and the proposed system with DR were compared in tunnels. When entering a tunnel, the position is only estimated from DR because the satellite signals is disconnected. Consequently, as the driving time increases, the positioning error increases. Additionally, the estimated positioning error varies with the accuracy of sensors used in estimation and the performance of the estimation algorithm. The average positioning error of the proposed system was up to over about 41.5% lower than it of model C in all sections. The results of the test can be summarized as follows:

- RTK has better performance than GNSS and GNSS/INS modules in dense urban environments. However, the number of receivable satellites and geometry change rapidly due to the positions of high-rise apartments and moving visible satellites for vehicle positioning. Consequently, Aiding sensors and the additive performance improvement method to adapt to changes in the environment are needed.
- In the proposed algorithms, the steering angle is used to compensate for the yaw rate of MEMS-IMU, and the velocity calculated from the 4-wheel RPM is used to supplement the velocity of MEMS-IMU. Consequently, the proposed algorithms have a more stable positioning performance than GNSS/INS (only IMU) in places that cannot receive the satellite signals such as tunnels.
- By using a fuzzy logic system that adopts adaptive measurement noise covariance over PDOP, the number of receivable satellites and difference between estimation and RTK measurement, the positioning error generated by entering and exiting tunnels is decreased.

Consequently, in dense urban environments, the proposed system has an average positioning error of 0.48 m, a maximum positioning error of 1.96 m, and an LLA of 94.05%. Also, in tunnel environments, there is an average positioning error of 1.15 m, a maximum positioning error of 4.05 m, and an LLA of 66.67%.

## 6. Conclusions

This paper presented a study on an adaptive Kalman filter design based on fuzzy-innovation for the improvement of vehicle positioning performance in a dense urban environment.

The proposed method is based on the EKF which adaptively applies the measurement covariance matrix according to the driving state of the vehicle and the fuzzy logic system. The proposed method corrects the yaw rate of the MEMS-IMU with the steering angle value obtained from the in-vehicle sensor and replaces the estimated velocity of the MEMS-IMU using the velocity calculated from the 4-wheel RPM. This reduces the estimation error in the position, velocity, and attitude of the MEMS-IMU. The fuzzy logic system with input of PDOP, the number of visible satellites, and the innovation of EKF directly reflects the performance change in RTK in a dynamic environment. By correcting the heading and position according to the driving state defined in this paper, it prevents the divergence of the filter due to initialization error or modeling error and improves the estimation performance of the filter.

In order to analyze the performance of the proposed FI-AKF positioning system in a dense urban environment, computer simulations were performed to compare the performance of the existing extended Kalman filter and the innovation-based adaptive extended Kalman filter. In addition, we conducted a performance comparison experiment with a commercial positioning system in a field test. Through each experiment, it was confirmed that the proposed FI-AKF system has a higher positioning performance than comparitive systems in dense urban environments.

## Figures and Tables

**Table 1:** Criteria and policy for driving states.

Driving State	Criterion	Policy
Stop	Velocity is less than 0.2 m/s.	Mostly RTK is lower than IMU/OBD.
		No change in heading
Start Up Straight	Velocity is over 0.2 m/s. Radius is greater than a threshold.	Heading can be adjusted.
Start Up Turning Left/Right		-
Straight	Start Up Straight state lasted for 1 s.	Sensors reliability is determined by dynamics of vehicles in driving status.
Turning Left/Right	Start Up Turning Left/Right state lasted for 1 s. Velocity is over 0.2 m/s. Until initialization is complete.	

**Table 2:** Fuzzy control rule.

(a) Fuzzy Control Rule 1				
*A*		PDOP		
		Bad	Not Bad	Good
# of S	Small	Bad	Bad	Bad
	Medium	Bad	Not Bad	Good
	Large	Not Bad	Good	Good
**(b) Fuzzy Control Rule 2**				
σ		Innovation		
		Small	Medium	Large
*A*	Bad	Medium	Medium	Medium
	Not Bad	Small	Small	Large
	Good	Large	Small	Medium

**Table 3:** IMU Specifications.

Quantity	MPU-9250 (Proposed)	BMI-160 (Ublox M8U)	Honeywell HG1700	Honeywell HG9900
**Grade**	Consumer	Consumer	Tactical	Navigation
**Accelerometer Errors**				
**Bias**	60 mG	40 mG	1 mG	0.025 mG
**Scale Factor**	3000 ppm	1000 ppm	300 ppm	100 ppm
**Gyroscope Errors**				
**Bias**	36 deg/h	7.96 deg/h	1 deg/h	0.003 deg/h
**Angle Random** **Walk**	0.6 deg/sqrt (hour)	0.13 deg/sqrt (hour)	0.125 deg/sqrt (hour)	0.002 deg/sqrt (hour)
**Scale Factor**	3000 ppm	1000 ppm	150 ppm	5 ppm
**Cost**	≤USD 10	≤USD 10	≤USD 10,000	≤USD 50,000

**Table 4:** Average and maximum position errors and lane-level accuracy in scenario 1.

		Road 1	Road 2	Road 3	Road 4	Road 5	Road 6	Road 7	Road 8	Total
Model A (GNSS)	Avg.	4.69	2.34	0.42	2.84	1.14	0.49	2.37	1.45	1.73
	Max.	56.58	18.37	3.20	10.56	22.58	1.37	12.68	4.43	56.58
	LLA	47.47	54.55	96.59	34.00	84.86	100.00	55.14	70.14	70.78
Model B (GNSS)	Avg.	0.77	2.00	0.58	2.02	0.80	0.54	0.78	0.55	1.06
	Max.	1.80	3.31	1.04	2.86	1.66	1.45	1.25	1.35	3.31
	LLA	96.99	9.62	100.00	27.51	90.09	100.00	100.00	100.00	75.48
Model C (GNSS/INS)	Avg.	0.72	1.22	0.80	1.03	2.21	1.42	0.51	0.74	1.01
	Max.	1.55	2.49	1.53	2.60	3.55	2.82	1.35	1.47	3.55
	LLA	98.28	74.11	97.69	76.92	20.90	54.22	100.00	100.00	81.04
RTK	Avg.	1.26	0.60	0.63	0.35	0.72	0.46	0.48	0.25	0.54
	Max.	2.15	3.05	1.22	0.88	2.19	3.01	1.42	1.31	3.05
	LLA	50.00	89.20	100.00	100.00	88.29	91.31	100.00	100.00	91.79
Proposed	Avg.	1.10	0.54	0.57	0.31	0.33	0.39	0.57	0.23	0.48
	Max.	1.94	1.96	0.83	0.90	0.71	1.79	1.74	1.43	1.96
	LLA	59.52	89.81	100.00	100.00	100.00	96.97	97.61	100.00	94.08

**Table 5:** Average and maximum position errors and lane-level accuracy on scenario 2 roads.

		Road 1	Road 2	Road 3	Road 4	Road 5	Road 6	Road 7	Total
Model A (GNSS)	Avg.	0.72	2.70	1.07	0.45	0.80	1.44	2.31	0.87
	Max.	15.27	8.07	6.34	2.84	3.61	5.33	5.46	15.27
	LLA	96.99	62.86	91.79	96.98	83.75	73.27	20.45	91.28
Model B (GNSS)	Avg.	2.58	0.33	0.33	0.43	0.62	1.10	1.12	1.51
	Max.	9.24	3.71	4.90	2.24	0.94	1.43	2.50	9.24
	LLA	47.54	93.27	98.30	97.32	100.00	72.61	83.70	71.72
Model C (GNSS/INS)	Avg.	4.66	1.61	0.82	0.61	0.94	1.26	1.88	2.79
	Max.	13.17	7.00	2.32	1.38	1.30	5.49	2.16	13.17
	LLA	0.49	75.00	98.31	100.00	100.00	100.00	0.00	45.30
Proposed	Avg.	0.32	0.26	0.37	0.29	0.25	0.73	0.28	0.34
	Max.	1.41	2.91	3.87	0.73	1.35	3.20	1.12	3.87
	LLA	100.00	98.20	98.31	100.00	100.00	97.52	100.00	99.42

**Table 6:** Average and maximum position errors and lane-level accuracy in scenario 2 tunnels.

		Tunnel 1	Tunnel 2	Tunnel 3	Tunnel 4	Tunnel 5	Tunnel 6	Total
Model C (GNSS/INS)	Avg.	6.16	1.36	0.44	2.99	1.39	0.84	2.77
	Max.	9.75	7.09	0.77	9.68	8.01	3.90	9.75
	LLA	0.34	80.30	100.00	14.44	91.13	93.13	43.89
Proposed	Avg.	0.83	0.98	0.29	1.37	1.55	0.87	1.15
	Max.	2.85	4.05	0.70	2.22	3.39	1.36	4.05
	LLA	79.33	86.19	100.00	50.08	44.96	100.00	66.67
